# Reliability and Identification of Aortic Valve Prolapse in the Horse

**DOI:** 10.1186/1746-6148-9-9

**Published:** 2013-01-11

**Authors:** Gayle D Hallowell, Mark Bowen

**Affiliations:** 1 School of Veterinary Medicine and Science (SVMS), University of Nottingham, Sutton Bonington Campus, Sutton Bonington, Leicestershire, LE12 5RD, UK

**Keywords:** Cardiology, Equine, Echocardiography, Repeatability, Reproducibility

## Abstract

**Background:**

The objectives were to determine and assess the reliability of criteria for identification of aortic valve prolapse (AVP) using echocardiography in the horse.

**Results:**

Opinion of equine cardiologists indicated that a long-axis view of the aortic valve (AoV) was most commonly used for identification of AVP (46%; n=13). There was consensus that AVP could be mimicked by ultrasound probe malignment. This was confirmed in 7 healthy horses, where the appearance of AVP could be induced by malalignment. In a study of a further 8 healthy horses (5 with AVP) examined daily for 5 days, by two echocardiographers standardized imaging guidelines gave good to excellent agreement for the assessment of AVP (kappa>0.80) and good agreement between days and observers (kappa >0.6). The technique allowed for assessment of the degree of prolapse and measurement of the prolapse distance that provided excellent agreement between echocardiographers, days and observers (kappa/ICC>0.8). Assessments made using real-time zoomed images provided similar measurements to the standard views (ICC=0.9), with agreement for the identification of AVP (kappa>0.8).

Short axis views of the AoV were used for identification of AVP by fewer respondents (23%), however provided less agreement for the identification of AVP (kappa>0.6) and only adequate agreement with observations made in long axis (kappa>0.5), with AVP being identified more often in short axis (92%) compared to long axis (76%).

Orthogonal views were used by 31% of respondents to identify the presence of AVP, and 85% to identify cusp. Its identification on both views on 4 days was used to categorise horses as having AVP, providing a positive predictive value of 79% and negative predictive value of 18%. Only the non-coronary cusp (NCC) of the AoV was observed to prolapse in these studies. Prolapse of the NCC was confirmed during the optimisation study using four-dimensional echocardiography, which concurred with the findings of two-dimensional echocardiography.

**Conclusions:**

This study has demonstrated reliable diagnostic criteria for the identification and assessment of AVP that can be used for longitudinal research studies to better define the prevalence and natural history of this condition.

## Background

Aortic valve prolapse (AVP) is a common echocardiographic finding that has been defined as downward displacement of cuspal material below a line joining the points of attachment of the aortic valve (AoV) leaflets [1], such that one or more cusps billows into the left ventricle during diastole. Only a few reports have described AVP in the horse, despite being commonly identified on echocardiograms. It has been identified with a reported prevalence of 20% in normal Thoroughbred or Standardbred racehorses [2] and secondary to a ventricular septal defect in one horse [3]. The significance of identification of AVP in any species is unclear; it was initially proposed that AVP was only identified in human patients that had underlying heart disease [1], but more recent studies have suggested that it may be an early sign of valvular ageing [4]. These contradictory findings regarding the significance of AVP in human patients leads us to question whether AVP is a variation of normal or representative of early disease in the horse.

In horses, AVP has previously been identified on two-dimensional (2D) right parasternal long axis echocardiographic views of the left ventricular outflow tract (LVOT) [2] whereas in human patients this is combined with an M-mode of the short axis view of the AoV to provide additional diagnostic information [5]. The M-mode findings are not however specific to AVP in humans [5,6] or in horses [7] and there are currently no agreed imaging criteria to identify AVP in the horse, although a qualitative grading system in humans has been reported [8]. Furthermore, there are no agreed imaging criteria to quantify AVP in any species, even though methods for quantification of mitral valve prolapse (MVP) has been reported in humans and dogs [9-11]. The development of a reliable technique for the identification and quantification of AVP in the horse is essential to create a tool that can be subsequently used to determine the prevalence and natural history of this condition in this species.

The aim of this study is to create reliable imaging criteria for the evaluation of AVP in the horse. This will be achieved by defining current identification methods amongst a group of cardiologists and then subsequently identify criteria that can be used for its identification. These data will be used to justify, optimise and develop assessment criteria for AVP, which will be compared to findings obtained using four-dimensional (4D) echocardiography. Finally the reliability of these criteria for AVP and related factors associated with the development in AVP will be evaluated.

## Methods

### Development of a consensus for the assessment of AVP

Opinions of a small group (n=15) of forerunners in equine cardiology were canvassed via electronic mail, using seven multiple choice questions and free text response enquiring about whether the individuals identified AVP on short or long axis views of the AoV or both, the number of cardiac cycles examined, how they ascertained which cusp was prolapsing and whether the severity of AVP was graded. In addition, they were asked about if, and how the appearance of AVP could be artefactually created (See Additional file 1).

### Optimisation of assessment criteria for the identification of AVP

Seven Thoroughbred and Thoroughbred cross horses aged 6.3±1.8 years, weighing 560±21 Kg with a body condition score (BCS) of 4–5 out of 9 [12] and free of pathological cardiac murmurs consistent with valvular regurgitation were examined on two occasions approximately one month apart. The horses were restrained in a stall with a headcollar and leadrope without sedation. Two small areas, approximately 5-8cm square, were clipped on the left and right thoracic wall caudal to the olecranon, and water and ultrasound coupling gel^a^ were liberally applied to provide appropriate contact. The animals were acclimatised for 5 minutes prior to echocardiographic examination or until the HR was less than 40 beats per minute.

**Figure 1:**
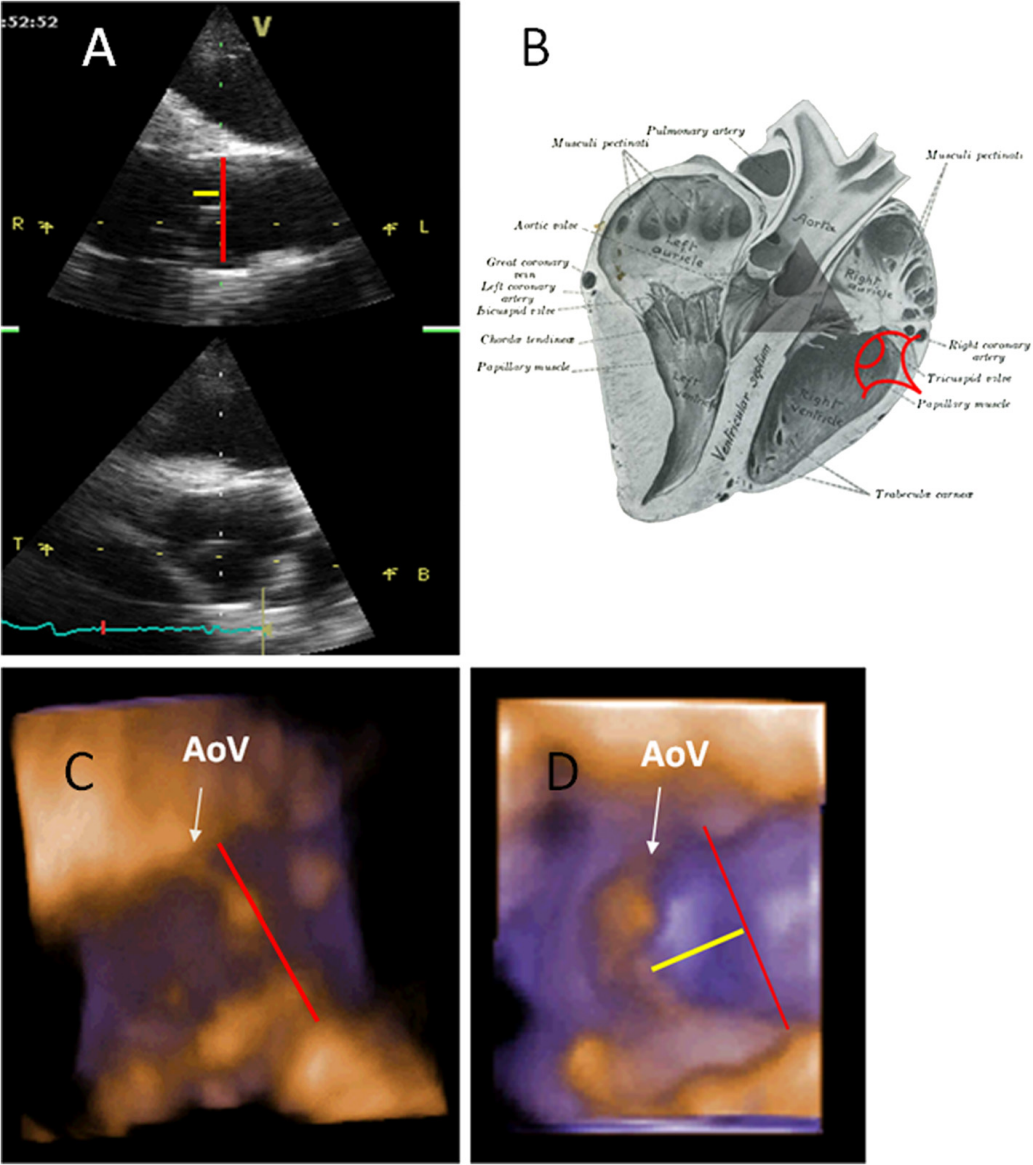
**A shows echocardiographic acquisition planes, of right parasternal long axis and short axis views through the aortic valve (AoV).B** depicts the visualisation plane used to reconstruct the three-dimensional images below (adapted from Sisson, 1914 [30]). **C** and **D** are reconstructed bronze-purple rendered three-dimensional echocardiographic images, which are digitally ‘dissected’ from the original image and were acquired using a 2.5MHz 3V phased-array transducer attached to Vivid 7 Dimension (GE Ultrasound, Bedford, UK) showing the appearance of the non-coronary cusp (NCC) of a normal AoV (**C**) and of a prolapsing NCC (**D**). The NCC is viewed from within the looking up. The white arrow on each diagram points towards NCC of the AoV, the red line joins the two coaptation points of the non-coronary cusp and the yellow line depicts prolapse of the cusp.

Right parasternal 2D long and short axis views of the LVOT and AoV and short axis view of the left ventricle (LV) were obtained using a phased-array transducer (2.5 MHz) attached to a portable ultrasound machine^b^ and on a second examination using both the portable machine^b^ and a 3V transducer (2.5 MHz) attached to Vivid 7 Dimension^c^ using simultaneous electrocardiography. Doppler interrogation of the AoV was undertaken using colour flow Doppler (CFD) to document the presence of physiological aortic valve regurgitation (PAR) [2]. Two-dimensional images were optimised so that normal or prolapsing valves could consistently be identified by both operators; this was done independently, but conducted on the same day. Images were recorded as cineloops (5 cardiac cycles) during periods of sinus rhythm and analysed offline. The presence of AVP was defined as bowing of any cusp of the AoV during diastole into the LVOT from a long axis view and apparent movement out of the visualisation plane in a short axis view. In animals without AVP, the imaging plane was changed to document whether AVP could be mimicked in non-standard views. Where possible, landmarks associated with the artefactual creation of AVP were recorded. During the second examination, all horses underwent echocardiographic examinations using the two ultrasound machines with the same protocol, to confirm agreement between images obtained from the different machines. Neither operator referred to previous findings or to the observations of the other operator. During the second examination, 4D echocardiography of the LVOT and AoV was undertaken using real-time translation to generate 4D cineloops that were stored for offline processing using dedicated software^d^. Four-dimensional constructs were rendered from different visualisation points and rotated to allow full evaluation of the AoV in multiple planes (Figure 1). These included short and long axis views of the AoV, views oriented from within the LVOT angled up towards the AoV and oriented from within the Ao angled down towards the AoV and also within the Ao angled up towards the NCC. Four-dimensional findings were compared to those from 2D images.

During assessment of each cineloop, images were graded in terms of quality; excellent quality images allowed clear, easy visualisation of all structures of interest, good quality images were as for excellent, but spontaneous contrast or lung obscured certain cardiac structures for some of the cardiac cycle, images were deemed to be of adequate quality when there was poor definition of some of the structures but measurements could still be performed and images were deemed poor when there were several structures that could not be easily visualised for much of the cardiac cycle. Cineloops of poor quality were not evaluated.

### Reliability of identification and assessment of AVP

Eight normal Thoroughbred or Thoroughbred Irish Draught cross horses (5 geldings and 3 mares) aged 14.5±2.6 years, weighing 628±86 Kg and with a BCS of 5-7/9 [12] were examined. All horses were free of pathological cardiac mur0murs consistent with valvular regurgitation. Horses underwent evaluation daily, for five consecutive days, as described from the optimisation study (Table 1) using a portable ultrasound machine^b^. All views were obtained using a standard technique [13,14] with the depth of penetration between 20 and 30cm, optimised for the view. Zoomed views were acquired in real-time focused on the AoV, with a scanning window of approximately 15cm. This technique creates a new image with an increased number of pixels to increase measurement accuracy [15]. Apart from imaging depth, power and gain, all other ultrasound parameters were consistent between animals.

**Table 1:** Criteria developed to consistently and repeatably identify AVP on right parasternal long and short axis cineloops of the left ventricular outflow tract (LVOT) and aortic valve (AoV)

**A) RIGHT-SIDED PARASTERNAL LONG-AXIS VIEW OF THE LVOT AND AOV**	**B) RIGHT-SIDED PARASTERNAL SHORT-AXIS VIEW OF THE AOV**
· Image obtained from RICS4	· Images obtained from the RICS4
· IVS perpendicular to the ultrasound beam	· AoV centred in the middle of the image with all three cusps visible
· Walls of the Ao parallel	· TV in the near field of the image
· Two AoV cusps visible	· PV in the far field of the image

Abbreviations: Ao – aorta; IVS – inter-ventricular septum; PV – pulmonary valve; RICS4 – right 4^th^ intercostal space; TV – tricuspid valve.

**Figure 2:**
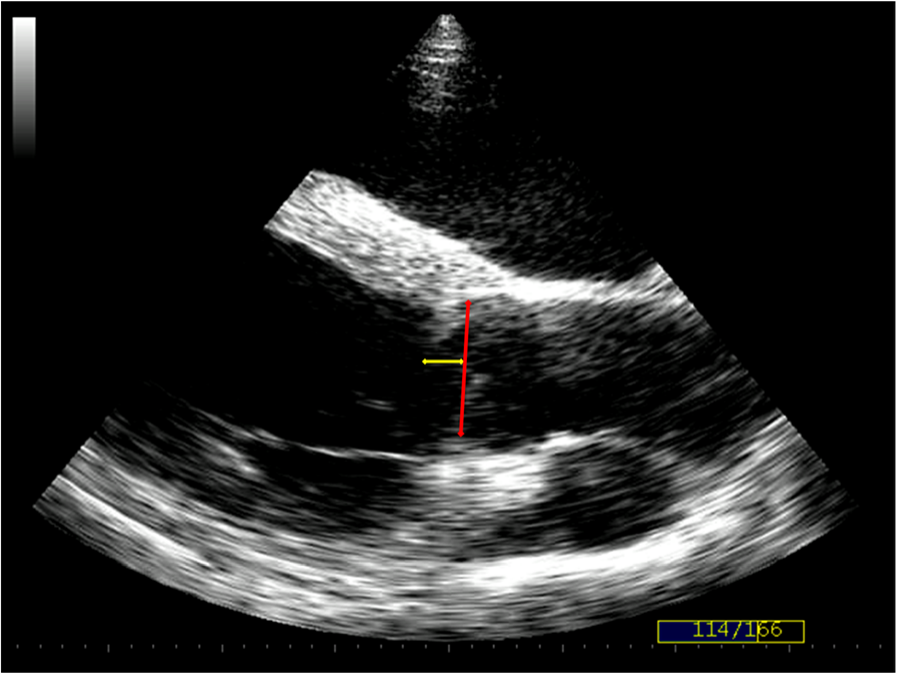
**Right parasternal long axis echocardiographic image of the left ventricular outflow tract showing the aortic valve in diastole.** The amount of aortic valve prolapse (AVP) was measured in centimetres from a line (yellow) perpendicular to one joining the attachment of the two leaflets (red). Cineloops of this view were also used to subjectively evaluate the severity of prolapse (mild, moderate or severe). The image above was obtained from a horse deemed to have mild AVP.

**Figure 3:**
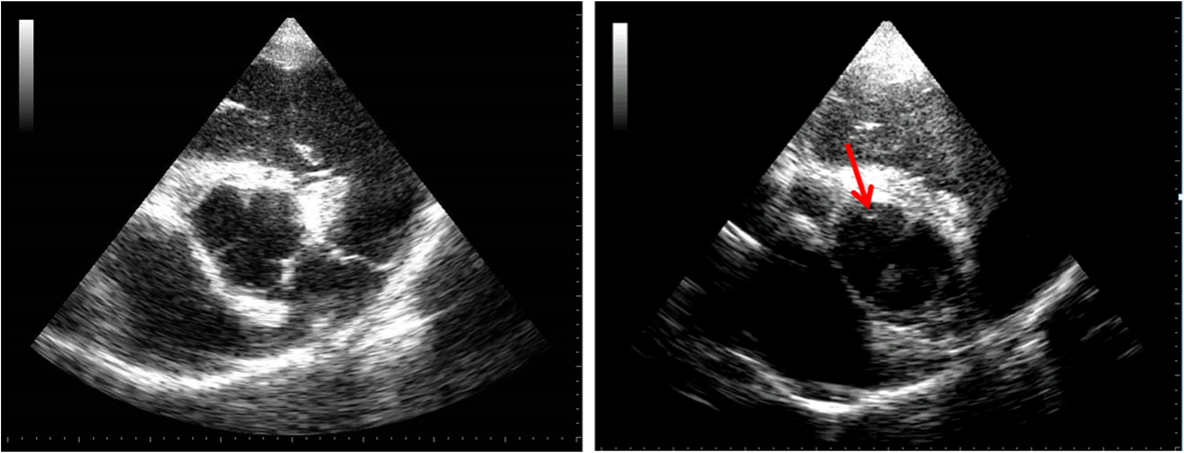
**Right parasternal short axis echocardiograms of the aortic valve (AoV). The image on the left shows a view of a normal AoV in the centre of the image as well as the landmarks described in the optimisation study.** The image on the right is from aortic valve prolapse (AVP) of the non-coronary cusp (NCC). The images shows the edges of the right and left coronary cusps and the red arrow is highlighting the NCC not being present within the imaging plane as the other two are.

Aortic valve prolapse was characterised over three consecutive cycles on both standard and zoomed views. Prolapse was identified from both long and short axis views (present or not present) and was further assessed using long axis views for qualitative assessment of degree of AVP (mild, moderate or severe) and quantitative assessment of amount of prolapse (cm). Mild prolapse was identified when less than a third of the cusp prolapsed into the LVOT in the latter half to third of diastole, moderate prolapse was identified when the whole cusp could be visualised prolapsing throughout diastole forming a slight curve and severe prolapse was identified when the cusp was visualised prolapsing throughout diastole forming an obvious ‘C’ shape. The amount of prolapse was measured in centimetres from a line perpendicular to one joining the attachment of the two leaflets on a right parasternal long axis view of the LVOT (Figure 2). Prolapse of any cusp was identified from short axis views of the AoV when the opposing edges of the cusps were seen to meet and then move out of the imaging plane during diastole, without any concurrent movement of other aspects of the cineloop (Figure 3).

In order to assess technique reproducibility (inter-echocardiographer variability), echocardiographic examinations were performed by both echocardiographers on one of the five days and one observer assessed the images. To assess technique repeatability (daily variability), one observer assessed images obtained by the same echocardiographer from each horse examined on the five different days. To assess measurement repeatability (intra-observer variability), one observer assessed all echocardiograms (n=48), and a second observer assessed a subset of thirty echocardiograms on three occasions at least one week apart without reference to previous records. This enabled repeatability to be assessed for each observer. To assess measurement reproducibility (inter-observer variability), measurements were compared from the thirty echocardiograms measured by both observers.

### Ethical review

This study was approved by the ethics and welfare committee of The School of Veterinary Medicine and Science, University of Nottingham.

### Statistical analysis

All data are displayed as mean (±SD) or expressed as a percentage. Continuous data was normally distributed. Reliability was assessed by comparing results from different observations of the same measure and by demonstrating concordance. Differences between observations from two echocardiographers (technique reproducibility), observers (measurement reproducibility) and measurements obtained from normal and zoomed views were determined using a paired Student’s T-test. Differences between observations taken from different days (technique repeatability) and from different observations (measurement repeatability) were determined by repeated measures ANOVA. Comparisons between observations on long and short axis views, agreement between long and short axis views and between normal and zoomed views were evaluated using a McNemar’s test. Fisher’s exact test was used to assess agreement regarding detection of AVP with image quality and the semi-quantitative assessment of prolapse. Concordance was determined by calculation of intra-class correlation coefficient (ICC), coefficient of variation (CV) and reliability coefficient (RC) [16] for quantitative measures for both repeatability and reproducibility. Cohen’s Kappa was used to assess agreement between categorical data (AVP presence and severity). Concordance (ICC and Cohen’s Kappa) was considered excellent when greater than 0.8, good when 0.6-0.8, adequate when 0.4-0.6 and poor when less than 0.4 [17]. All statistical analyses were undertaken using commercial statistical packages^e,f^. Significance was assumed when p<0.05.

## Results

### Development of a consensus for the assessment of AVP

Responses were received from 87% (13/15) of those questioned. The most common, single view used for the identification of AVP was a long axis view of the LVOT (46%), whilst fewer used a short axis view alone (23%). Thirty one per cent of respondents used both long and short axis views to identify AVP, although 46% indicated that they could only confirm the presence of AVP when visualised on both views, with the remainder making this assessment using one view alone. In order to assess which cusp was affected the majority (85%) used both views, whilst 15% used a single view. All respondents indicated that AVP needed to be visualised on 3–5 consecutive cycles for it to be positively identified and all assessed severity qualitatively. All responded that AVP could be artefactually created, and this could be created by triangulation of the LVOT (77%), when the IVS was angled downwards (31%) or on any cineloop that deviated from a standard view (23%).

### Optimisation of assessment criteria for the identification Of AVP

Five of the seven horses had left-sided murmurs consistent with ejection murmurs and all of the horses had normal resting heart rates (HR; 35±4.5BPM). Echocardiographic examination revealed normal cardiac dimensions [13,14,18] and no horses had structural valvular pathology. Image quality was deemed excellent in six of the seven horses and good in one horse. Four horses had AVP affecting the NCC, whilst the other three horses had functionally normal AoV. Five of the seven horses (four with AVP and one with a normal AoV) had PAR. There was 100% agreement between observers, examination days and machines for the presence of AVP. Prolapse was also observed affecting the NCC of affected horses using 4D echocardiography, whilst other cusps appeared normal. Prolapse was easier to visualise using 4D echocardiography from transverse views, where the viewpoint was set within the LVOT, observing the ventricular surface of the AoV, compared to an aortic viewpoint, observing the aortic surface.

Both operators were able to create the appearance of AVP in those horses with normal valve function from long axis views when there was triangulation or convergence of the walls of the Ao (n=3/3; beam transecting the Ao obliquely), when the IVS was angled downwards (n=2/3) and when a large amount of artefactually thickened IVS was visible adjacent to the AoV (n=2/3; beam transecting the IVS obliquely). For each of these variations, the appearance of prolapse was associated with either the cusps on the right of the aorta (Ao); either the NCC or right coronary cusp (RCC). Echocardiographic imaging criteria were developed to ensure consistent imaging for the reliable identification of AVP (Table 1).

### Reliability of identification and assessment Of AVP

Three of the eight horses had left-sided murmurs consistent with ejection murmurs and all of the horses had normal resting HRs (37.5±3.7BPM). Echocardiographic examination revealed normal cardiac dimensions [13,14,18] and no horses had structural valvular pathology. Image quality was excellent in 66% of cineloops (n=396), good in 25% of cineloops (n=151) and adequate in 7% of cineloops (n=41). Thirty-six cineloops were excluded from further analysis as they could not be assessed.

Five of the eight horses were found to have AVP, all affecting the NCC, present on multiple (≥4) examinations identified on both short and long axis views. The other three horses examined had functionally normal AoV. Overall, AVP was qualitatively assessed as mild (n=2), moderate (n=2) and severe (n=1) in the five affected horses, with a measured prolapse distance of 1.77±0.87cm, based on their appearance on at least 4 examinations. Seven of the eight horses (five with AVP and two with normal AoV) had PAR. Using the presence of AVP on both long and short axis views on 4 or more examinations as the ‘gold-standard’, the positive predictive value for determination of AVP was 79.3% and negative predictive value was 18.2%.

### Technique reproducibility (inter-echocardiographer variability)

There was excellent agreement for the identification of AVP from observations between both echocardiographers when interrogating the right parasternal long axis view of the LVOT (92%; kappa=0.82; p=0.63) and good agreement when assessing the short axis view (87.5%; kappa=0.76; p=0.46). Image quality affected the agreement regarding AVP identification from different echocardiographers’ images (p=0.03). The agreement between adequate quality images (72%) was less than for the good (89%) and excellent (94%) quality images, but there were no differences between good and excellent quality images. There was excellent agreement regarding assessment of the degree of prolapse (kappa=0.84; p=0.59) and quantification of AVP (echocardiographer 1: 1.84±0.48 cm, echocardiographer 2: 1.80±0.50 cm; ICC=0.91; p=0.79; CV=4.5%; RC=0.42 cm). There were no differences in measurements of AVP made between normal and zoomed views (normal=1.84±0.48, zoomed=1.83±0.38, ICC=0.90; p=0.76; CV=10%; RC=0.39 cm). Similarly, there was excellent agreement between normal and zoomed long axis views (96%; kappa=0.89; p=0.78) and good agreement between short axis (89%; kappa=0.78; p-0.52). There was less agreement between the identification of AVP on a long axis view compared to a short axis view of the LVOT (84%; kappa=0.52; p=0.32), where AVP was more frequently identified on a short axis view (long axis: 76% of horses; short axis 92% of horses).

### Technique repeatability (daily variability)

There was good agreement between days for the identification of AVP (87.5%; kappa=0.64; p=0.53) with AVP not being observed on one day in four horses that were classified as having AVP. The appearance of AVP was present on one day in one horse that was otherwise considered to have normal AoV function. There was excellent agreement between AVP distance measurements between days (ICC=0.86; p=0.55; CV=6.0%; RC=0.44 cm).

### Measurement repeatability (intra-observer variability)

Agreement regarding AVP identification between observations was 96% (p=0.88) and 92% (p=0.82) for the two observers. Discrepancy between AVP identification arose only for images of adequate quality. Repeatability of the assessment of the degree of prolapse was good (p=0.45 and p=0.62). There were no differences in any of the repeated observations (measurements) of AVP distance made by either observer and therefore the data was pooled and provided excellent agreement (measurement 1: 2.02±0.60, measurement 2: 1.95±0.56, measurement 3: 1.95±0.54; ICC=0.90; p>0.50; CV= 3.6%; RC=0.22 cm).

### Measurement reproducibility (inter-operator variability)

There was good agreement for the identification of AVP between observers from both long axis (90%; kappa=0.78, p=0.58) and short axis (83%; kappa=0.73; p=0.42) views of the AoV and LVOT. As with technique reproducibility, image quality affected AVP identification between the two observers; agreement between good (84%) and excellent (90%) quality images was better than adequate ones (69%; p=0.01). There was excellent agreement for the degree of AVP (89%; p=0.68) and amount (observer 1: 1.90±0.42, observer 2: 1.85±0.45; ICC=0.82; p=0.58; CV=3.38; RC=0.15 cm).

## Discussion

AVP was a common finding in both studies in a variety of horse types in agreement with previous observations [2]. To date, there have been no anatomical or echocardiographic landmarks reported for reliable identification of AVP in any species, despite the fact that the appearance of AVP in human patients can be artefactually created if the AoV is bisected by the ultrasound beam at an oblique angle [19]. This study has documented clear imaging criteria to ensure consistency of echocardiographic acquisition for the identification of AVP. Furthermore, these criteria allow for the reliable detection, assessment and measurement of AVP in the horse when applied to multiple cardiac cycles from the same animal. Whilst AVP may not represent a clinically important condition in its own right, to further understand the mechanisms and natural history of AVP an accurate method is required to monitor changes over time or with pharmacological intervention. Image quality affected the reliability of identification and assessment of AVP, which is supported by a previous study evaluating MVP identification [20], where there was an improved correlation between better quality images and highlights the need for the highest quality of image when interpreting echocardiograms.

The consensus opinion of equine cardiologists was that AVP could be artefactually created by incorrect probe angulation. Although this has been reported in humans [19], this had not previously been described in horses. The image optimisation study concurred with the consensus opinion and clearly demonstrated that a failure to bisect the Ao perpendicularly could result in the appearance of AVP in horses that were shown to have normal valve function. By using the criteria outlined in the image optimisation study it is possible to create consistent views of the Ao that enables reliable identification of AVP as demonstrated by evaluating technique reproducibility. Despite this, the appearance of AVP may still be apparent in a small number of horses that have normal valve function when examined on a single occasion, as demonstrated by the negative predictive value reported in this study. The reason for this small number of ‘false-positives’ is unclear, although it is known that the AoV is under autonomic and neurohormonal control [21-24] and therefore it is possible that valve function does change from day-to-day; indeed the largest variability arose between days, rather than between operators or echocardiographers. As such, horses were only definitely categorised as having AVP based on the appearance on multiple occasions. Therefore to definitively diagnose AVP, multiple examinations should be performed.

There was no consensus as to whether long or short axis views were most appropriate for the identification of AVP. The only published report of AVP identification in horses used a long axis view [2], and this was also commonly used. Indeed, although most respondents used both views to identify the affected cusp, there was a discrepancy between the number who used two views to identify AVP and the number requiring two views to be confident in this assessment. This finding is to be expected since there are no published studies assessing repeatability of identification of AVP in any species, no consensus diagnostic criteria for AVP in human patients and no diagnostic ‘gold-standard’ that can be applied for evaluation of AoV function. Robust criteria were therefore essential in order to validate these methods, and thus a horse was only defined as having AVP on each examination when this could be visualised on orthogonal views. This resulted in good identification of AVP as demonstrated by the high positive predictive value. The identification of AVP from long axis views was more repeatable and reproducible than from short axis views. Aortic valve prolapse was identified more often from a short axis view, which may suggest over interpretation in view of the poorer agreement. These findings suggest that the image interpretation is clearer when looking for apical movement of the valve cusp in long axis, rather than from a short axis view. Therefore the long axis view, the single most commonly used by the equine echocardiographers, is likely to more reliable when using only a single view for its identification. There was better reproducibility than reported for MVP identification in humans and dogs [25,26]. This likely relates to operator experience, where both observers had been involved in the development of the definition for AVP and had an interest in this condition. This is supported by studies of MVP, where reproducibility is affected by operator experience [25,26].

There was agreement from equine echocardiographers that to identify which valve cusp prolapsed, orthogonal views were required. These criteria were applied in this study, although in long axis it was not possible to differentiate between the RCC or the NCC of the AoV and therefore limited information regarding individual cusp could be obtained from these views. Since AVP of the left coronary cusp was not observed in this study it is questionable what further benefit is obtained in relation to the specific identification of valve cusps in animals with AVP from a long axis view. Indeed, in short axis, only the NCC was observed to prolapse. This contradicts studies in humans, where the RCC is most commonly affected [1,4,19]. Further study in other breeds and types of horse is warranted to confirm whether this observation is representative of all horses. If naturally occurring AVP only affects the NCC in the horse, then orthogonal views would remain indicated as part of its assessment, since prolapse of other valve cusps may represent different mechanisms.

Qualitative assessment of AVP severity was undertaken by all respondents, but no comments were made regarding how this was assessed, and thus far this technique has not been reported in AVP in horses. A semi-quantitative system has been described in human patients [8] and this was adapted for the current study. There was excellent reliability for the qualitative assessment of AVP suggesting that this may be a reliable and simple approach to monitoring changes in the amount of AVP, thus validating the criteria developed. Quantitative assessment of AVP was not undertaken by any respondent, which is expected since no criteria have been established for the quantitative measurement in any species. A technique used for the measurement of MVP was adapted for this study [10,27,28] and provided better reliability than the previously published grading system for MVP [25]. It is proposed that these differences in reliability are due to improved definitions of each grade and the simplicity of the AoV structure compared to the mitral valve. Both observers in the current study may have also been more familiar with each grade compared to the previous study. Assessment of reliability in a group of cardiologists of different experience could be used to further validate these definitions.

Whilst respondents did not comment further about other techniques used for the identification or assessment of AVP, the zoom facility was utilised since it was thought that it would provide better image quality. It was used to allow for improved assessment of valve function as it focused upon a particular structure, increasing resolution and is commonly used in human echocardiography [29]. This had no impact on the ability to identify or quantify AVP. As such it is concluded that zoomed image echocardiography provides no additional benefit for the assessment of AVP in the horse. Although 4D echocardiography has not been validated for use in the horse, it was used in this study to confirm the presence and identify the cusp affected by AVP by visualising apical movement of the cusp in concurrent orthogonal 2D images and by rendering these into an image that could be visualised from the ventricular surface. Although 4D echocardiography is not a gold standard, it does allow a real-time construct of the area of interest, which is of great value for confirming identification of a phenomenon that can only be identified *in situ*.

## Conclusions

Criteria have been developed for the accurate assessment of AVP in the horse from views of equine cardiologists. These criteria require high quality echocardiographic images, with consistent landmarks to ensure consistency of image acquisition. Having obtained consistent images, the identification, qualitative and quantitative assessment of AVP in the horse is reliable between echocardiographers, observers, and days. Long axis images provide the most useful information regarding identification and assessment of the degree of prolapse, although short axis views can be helpful to confirm which cusp is involved. This study is the first to identify appropriate assessment criteria that will allow further evaluation of AVP.

## Endnotes

a. Aquasonic gel 100, Parker Labs Inc., Fairfield, NJ, USA; b. MyLab30, Esoate, Genova, Italy; c.GE Ultrasound, Bedford, UK; d.Echopac™, GE Ultrasound, Bedford, UK; e. SPSS for Windows 15.0, SPSS Inc, Chicago, Illinois, USA; f. GraphPad 4.0, Graphpad Software, La Jolla, California, USA.

## Abbreviations

ANOVA: Analysis of variance; Ao: Aorta; AoS: Diameter of the aortic sinus; AoD: Aortic diameter; AoV: Aortic valve; AVP: Aortic valve prolapse; BCS: Body condition score; CFD: Colour flow Doppler ultrasound; ET: Ejection time; FS: Fractional shortening; FWd: Diameter of the free wall in diastole; FWs: Diameter of the free wall in systole; HR: Heart rate; ICC: Intra-class correlation coefficient; IVS: Inter-ventricular septum; IVSd: Diameter of the inter-ventricular septum in diastole; IVSs: Diameter of the inter-ventricular septum in systole; LV: Left ventricle; LVDd: Diameter of the left ventricle in systole; LVOT: Left ventricular outflow tract; MVP: Mitral valve prolapse; PAR: Physiological aortic regurgitation; PV: Pulmonary valve; RCC: Right coronary cusp; TV: Tricuspid valve.

## Competing interests

The authors declare that they have no competing interests.

## Author’s contributions

GH and MB both heavily involved in study design, acquisition and analysis of images and manuscript writing; GH additionally undertook statistical analysis. Both authors read and approved the final manuscript.

## Supplementary Material

Additional file 1 Question sent to a group of equine cardiologists. Click here for file
