# Supplementary material for: Reliability and Identification of Aortic Valve Prolapse in the Horse
Source: BMC Vet Res. 2013 Jan 11;9:9. doi: 10.1186/1746-6148-9-9 (PMC3547808; doi:10.1186/1746-6148-9-9)
Supplement: Additional file 1 — Question sent to a group of equine cardiologists. [file 1746-6148-9-9-S1.docx]

**ADDITIONAL FILE 1: Question sent to a group of equine cardiologists**

1. Do you diagnose AVP on short axis or long axis views of the left ventricular outflow tract?

Short Long Do not diagnose

1. Does prolapse have to be observable on both short and long axis views or is one sufficient?

Single View Multiple views

1. Do you ascertain which cusp is prolapsing from short, long or both views of the left ventricular outflow tract from a right parasternal orientation?

Short Long Both

1. How many cardiac cycles do you need to visualise AVP on to make a diagnosis?

1 3 5 Other______________

1. Do you think AVP can be created as an artefact by not having a standard long axis view of the LVOT?

Yes No Hadn’t thought about it

1. If Yes: Which of the following do you think could create the appearance of AVP
   1. Cranially rotated so that the walls of the aorta are not parallel.
   2. Sternally angled so that the septum is angled downwards
   3. Other
2. Do you currently use a method to quantify AVP severity and if so how?

No Qualitative Quantitative

If quantify, how do you do this? _________________________________

Any other thoughts or comments would be gratefully received and acknowledged

Additional File 1: Questions distributed via electronic mail to a group of fifteen equine cardiologists to ascertain their thoughts regarding the diagnosis of aortic valve prolapse (AVP) in the horse. Abbreviations: LVOT: left ventricular outflow tract
